# Sugar–Lectin Interactions for Direct and Selective Detection of *Escherichia coli* Bacteria Using QCM Biosensor

**DOI:** 10.3390/bios13030337

**Published:** 2023-03-03

**Authors:** Gaddi B. Eshun, Heather A. Crapo, Idris Yazgan, Lauren Cronmiller, Omowunmi A. Sadik

**Affiliations:** 1 Chemistry and Environmental Science, New Jersey Institute of Technology, University Heights, Newark, NJ 07102, USA; 2 Department of Chemistry, Center for Research in Advanced Sensing Technologies & Environmental Sustainability (CREATES), State University of New York at Binghamton, Binghamton, NY 13902, USA

**Keywords:** quartz crystal microbalance, sensor, bacterial enumeration, ligands, *E. coli*, FimH, selectivity

## Abstract

Pathogenic *Escherichia coli* (*E. coli*) remains a safety concern in the preservation and quality of green leafy vegetables. Sugar–lectin interactions provide a reliable, specific, and effective sensing platform for the detection of bacteria as compared to the tedious conventional plate counting technique. Herein, we present the synthesis of 4-(N-mannosyl) benzoic acid (4-NMBA) and 4-thiophenyl-N-mannose (4-TNM) via a two-step reductive amination for the detection of *E. coli* using a quartz crystal microbalance (QCM) biosensor. The 4-NMBA was synthesized with mannose and para-aminobenzoic (4-PBA), while the 4-TNM was synthesized with mannose and 4-aminophenyl disulfide (4-AHP) using water and acetic acid in a 1:1 ratio. The resultant structure of mannose derivatives (4-NMBA and 4-TNM) was characterized and confirmed using analytical tools, such as Mass Spectrometer, SEM, and FTIR. The choice of ligands (mannose derivatives) is ascribed to the specific recognition of mannose to the FimH lectin of the type 1 pilus of *E. coli*. Furthermore, the 4-PBA and 4-AHP conjugated to mannose increase the ligand affinity to FimH lectins. The setup of the QCM biosensor was composed of modification of the crystal surface and the covalent attachment of ligands for the detection of *E. coli.* The piezoelectric effect (frequency shift of the quartz) was proportional to the change in mass added to the gold crystal surface. Both the 4-NMBA- and 4-TNM-coated QCM sensors had a limit of detection of 3.7 CFU/mL and 6.6 CFU/mL with a sensitivity of 2.56 × 10^3^ ng/mL and 8.99 × 10^−5^ ng/mL, respectively, within the dynamic range of 10^3^ to 10^6^ CFU/mL. This study demonstrates the application of ligand-coated QCM biosensors as a cost-effective, simple, and label-free technology for monitoring pathogenic bacteria via molecular interactions on crystal surfaces.

## 1. Introduction

Pathogenic bacteria pose a threat to food quality and water systems globally. *E. coli* is a commensal microorganism that naturally inhabits the intestinal tracts of warm-blooded animals to facilitate vitamin B12 synthesis [1]. Shiga toxin-producing *E. coli* (STEC) is an infectious strain of *E.coli* that causes pediatric hemolytic uremic syndrome (HUS) and acute gastroenteritis by the consumption of contaminated food and water [2,3]. The case of the hemolytic uremic syndrome and diarrhea outbreak in May 2011 was linked to *Escherichia coli* O104:H4, which recorded 810 cases and 39 deaths [4]. Furthermore, 9.4 million illnesses and 1351 deaths annually in the United States are attributed to foodborne infections. For instance, <1 CFU/mL of *E. coli* can cause detrimental effects in humans [5,6]. These alarming numbers call for researchers and healthcare practitioners to investigate and develop sensitive methods for detecting the presence of *E. coli* in contaminated food samples and water. The goal of this study is to design a mannose derivative for the detection of *E. coli* via a QCM biosensor. 

Bacteria toxins bind to the carbohydrate derivatives present on cell surfaces. The adherence and binding affinity of bacteria to human cells is the beginning of their pathogenesis [7]. The attachment of bacteria to cells provides the basis for developing a biosensor that targets bacteria. For example, FimH is type 1 pili expressed in *E. coli* that binds to mannose [8,9]. Cumulative reports have shown that introducing hydrophobic groups and phenyl rings to mannose enhances the affinity between mannose and FimH lectin [8,10]. This idea is utilized in the design of biosensors. Conventional methods to detect and quantify foodborne pathogens in water systems and food products depend on reliable techniques and trained expertise. Furthermore, these instruments are time-consuming and function within a specific detection range. However, fluorescence [11], ELISA [12], and electrochemistry [13] are a few biosensors applied to rapidly detect foodborne pathogens [14]. The limitations of these detection methods increase the need to develop new rapid, reliable, portable, reagent-less, field-deployable, and inexpensive sensors. 

Quartz crystal microbalance (QCM) is one method that has the potential for rapid and inexpensive detection [15]. However, low detection limits of bacteria using QCM biosensors is a challenge partly due to the sample size, and the resultant sensitivity is compromised by the interference of the matrix effect [15,16]. QCM is an acoustic (mass) piezoelectric sensing technology that detects minute changes in mass (ng, nanogram level). The change in mass of the analyte on the quartz surface is proportional to the oscillating frequency shift of the quartz, and this phenomenon is known as the piezoelectric effect [17,18]. In this regard, QCM sensing permits cost-effective, real-time monitoring of molecular interactions between sugar and lectin on a gold crystal surface. Notably, several QCM sensors have been developed to target proteins and viruses. Reports by Mao et al. [19] show the application of QCM biosensors to detect *E. coli*. The sensor was prepared with ssDNA probes complementary to a target DNA combined with nanoparticle amplification [19]. The work was completed by conjugating the DNA sequences to Fe_3_O_4_ nanoparticles via streptavidin to increase the frequency change with promising detection limits and sensitivity [19,20].

In this work, we demonstrated the synthesis of mannose-derived ligands (4-NMBA and 4-TNM have) for detecting *E. coli* via interaction with the FimH lectin, using QCM as a detection platform. First, 4-NMBA was synthesized using mannose and para-aminobenzoic acid, while the 4-TNM was synthesized using mannose and 4-aminophenyl disulfide [8]. The ligands were characterized using a mass spectrometer, SEM, and FTIR. Using the Sauerbrey equation [21,22], we could calculate the different masses of bacteria added to the mannose-derived coated gold crystal. This is the first study demonstrating the application of a QCM-based biosensor utilizing sugar–lectin interactions to directly detect bacteria using 11-mercaptoundecanoic (MUA) as a linker to attach the sugars to the gold-plated crystal surface. Our results showed low detection limits and low sensitivity. This study broadens the cost-effective and reliable methods available for detecting pathogenic bacteria. 

## 2. Materials and Methods

### 2.1. Materials

D (+)-mannose, 11-mercaptoundecanoic acid(MUA), 4-aminophenyl disulfide, and tryptic soy broth were purchased from Sigma (St. Louis, MO, USA). Para-Aminobenzoic acid and N-(3-dimethyl aminopropyl)-N′-ethyl carbodiimide (EDC) were purchased from Fisher Scientific (Fair Lawn, NJ, USA). The borane–dimethylamine complex was purchased from Acros Organic (Beel, Belgium). N-hydroxysuccimide (NHS) was purchased from Pierce Chemical Company (Waltham, MA, USA). *Escherichia coli* (25922) were purchased from ATCC (Manassas, VA, USA). A quartz crystal analyzer model QCA917 from Seiko EG&G was used for all measurements. Furthermore, 10 MHz quartz crystals plated with gold were purchased from Princeton Applied Research (Berwyn, PA, USA.) All reagents were analytical grade. Aqueous solutions were prepared with nano-pure water with 18.2 MΩ specific resistance.

### 2.2. Synthesis of Mannose-Derived Ligands

Mannose derivative (4-NMBA) was synthesized by reacting the aldehyde group of D (+)-mannose and the amino group of para-aminobenzoic acid (reductive amination). Briefly, 20 mM of D (+)-mannose was dissolved in a 1:1 ratio of ultrapure water and acetic acid. Once completely dissolved, 22 mM of p-aminobenzoic acid was added to the reaction. The solutions were mixed and incubated in a sealed flask for 1 h at 20 °C. Amination of the reactants was achieved following this step. After incubation, a 50 mM borane–dimethylamine reducing agent was added to the reaction and incubated overnight to achieve the reduction step. The resulting product was filtered and washed with pure water to remove unreacted reagents. The final product was crystallized. A similar procedure was adapted for synthesizing 4-TNM using 4-aminophenyl disulfide, for which 11 mM concentration was utilized. The names and identities of the synthesized ligands were predicted using ChemDraw and confirmed with mass spectrometry [8].

### 2.3. Characterization of Mannose-Derived Ligands

Mass spectrometry characterization was performed for the mannose ligands to confirm the molecular structures. Both ligands were dissolved in liquid chromatography (LC) grade 50:50 mixture of acetic acid and water, followed by ESI-MS/MS analysis (Thermo Scientific LCQ, Fleet mass spectrometer) [8,23] to determine their molecular weights. The surface chemistry of mannose-derived ligands was visualized with scanning electron microscopy (SEM) performed at room temperature using JEOL SEM-2100F. Elemental analysis was determined by an energy-dispersive X-ray (EDX) analyzer. Fourier-transform infrared (FT-IR) spectra were recorded using a Shimadzu IRTracer-100 equipped with an ATR [24]. NMR characterization of the mannose derivatives was performed to determine the formation of a new amide bond between the mannose and the 4-PBA,4-AHP. 

### 2.4. Fabrication of Mannose-Derived QCM Sensor

**Scheme 1:**
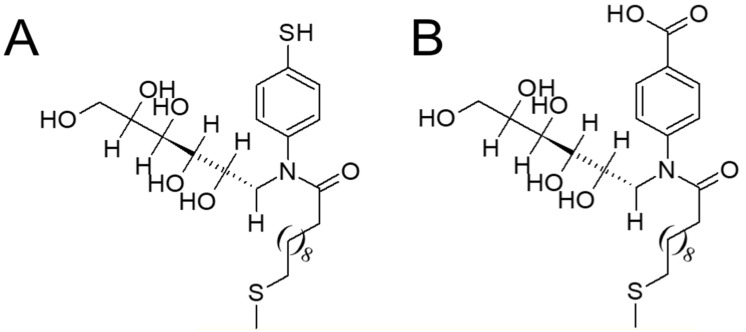
Schematic representation of immobilized MUA and mannose derivatives on gold quartz crystal. (**A**) 4-TNM (**B**) 4-NMBA.

To design the mannose-derived QCM biosensor, the gold crystal was cleaned following a standard procedure, modified with the mannose derivatives or *E. coli*, and inserted into a cassette connected to the quartz crystal analyzer. Both frequency shifts of the sensor were measured as a function of time. The sensor fabrication consisted of the following steps: a 1 mM solution of 11-mercaptoundecanoic acid (MUA) was made using ethanol 200 proof. Gold-plated quartz crystals were incubated in the MUA solution overnight. After incubation, the gold crystals were rinsed with ethanol to remove excess MUA [25]. Carboxyl groups of MUA were then activated using EDC and NHS solution mixture in the ratio of 0.3 to 0.1 respectively. The crystal was then coated with a 10 mM solution of the modified mannose ligand for 1 h and rinsed with ultrapure water. The proposed chemistry of mannose-based ligands is depicted in Scheme 1. The unbound *E. coli* to the modified gold crystal was removed by incubating the sensor in 100 mM glycine solution. After each modification step, the crystal was inserted into the cassette for QCM measurements to determine the amount of individual mass added to the crystal.

### 2.5. Bacteria Sample Preparation

Tryptic soy broth (TSB) (30 g/L) was prepared by following the manufacturer’s instructions. *E. coli* were grown in TSB at 37 °C for 18 h. The bacteria cultures were centrifuged at 4000 rpm for 15 min, the supernatant was discarded, and the precipitates were dispersed in 0.1 M phosphate buffer (pH 7.02) [26]. This procedure was repeated to obtain a purified *E. coli* analyte. The bacteria concentration was adjusted with McFarland standards to achieve the desired concentration for optimal QCM detection. Subsequent serial dilution was performed to achieve the different concentrations of bacteria tested. Bacteria suspensions with a concentration of 10^3^ to 10^6^ CFU/mL were used as the samples. 

### 2.6. Bacteria Detection Using QCM Immobilized Mannose Derivatives

**Scheme 2:**
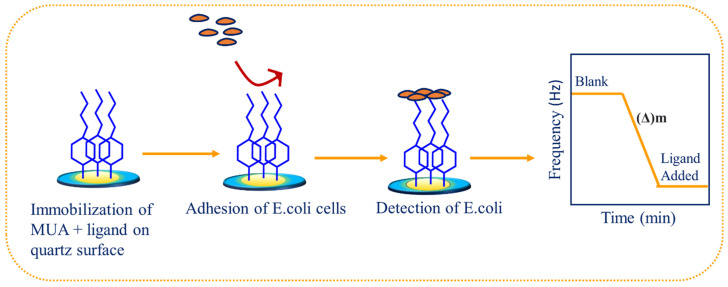
Schematic steps showing the detection of *E. coli* via mannose-derived QCM biosensor.

*E. coli* on the QCM sensor surfaces was achieved via a micro-contact between the *E. coli* cells and the gold surfaces immobilized with mannose derivatives. The procedure entails pipetting an aliquot of 100 mL *E. coli* onto the modified gold crystal surface. The set-up was allowed to dry under nitrogen gas. The crystal was rinsed with ultrapure water to remove any unbound bacteria. The crystal was inserted into the cassette, and the change in frequency before and after the addition of the bacteria was measured (Scheme 2). The difference in mass was determined by using the Sauerbrey equation [22].

### 2.7. Statistical Analysis 

Experiments were performed in three replicates (*n* = 3). The mean and standard deviations were calculated for each triplicate of masses. The differences between the mean were subjected to a one-way analysis of variance and t-test, and *p* < 0.05 was significant.

## 3. Result and Discussion

### 3.1. Synthesis of Mannose Derivatives 

The synthesis of 4-NMBA was achieved by reacting para-aminobenzoic acid with mannose, while 4-TNM was formed from the reaction between mannose and 4-aminophenyl disulfide (6-AHP) following a revised protocol published elsewhere [8]. The choice of these ligands is based on the idea that hydrophobic (long-chain aliphatic groups) and phenyl rings attached to mannose enhance their affinity and selectivity towards FimH lectin. The resultant reaction products were white crystalline powders that were purified using suction filtration and characterized using analytical devices to confirm their morphology and structures. The aim of this study was mainly to develop a QCM biosensor that determines frequency changes when the mannose derivative binds to pili 1 FimH lectin of *E. coli*. The applied mass determines the frequency shift of the gold quartz on its surface according to the Sauerbrey relationship. 

### 3.2. Mass Spectrometry Characterization 

**Figure 1:**
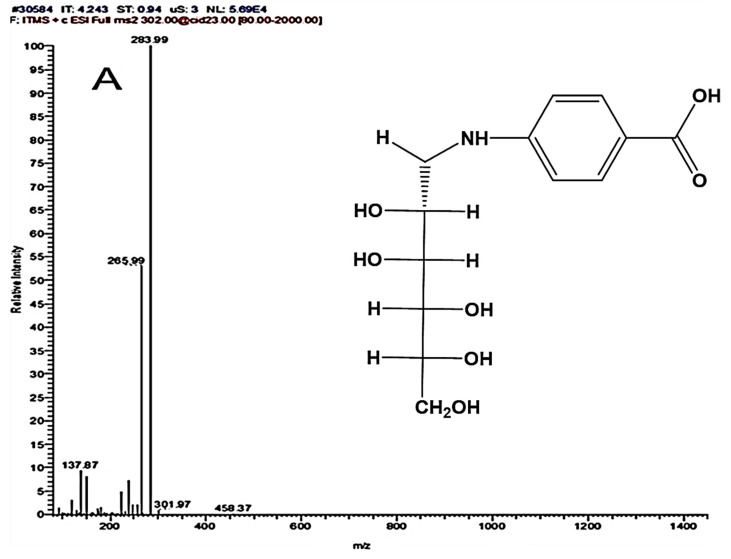

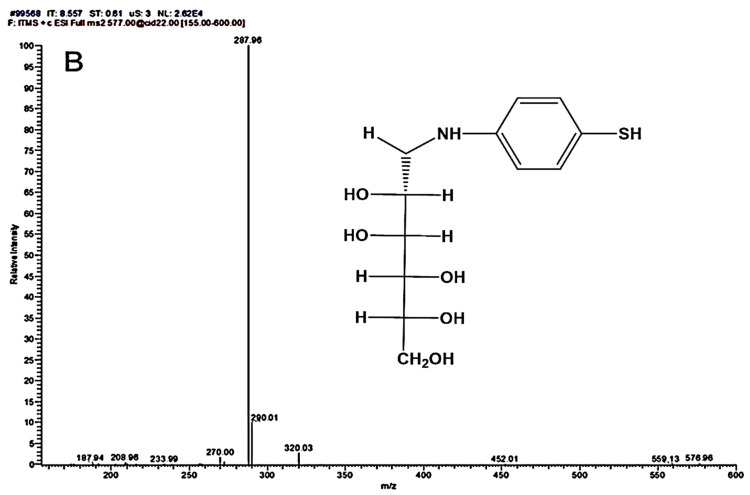
MS/MS of mannose derivatives (**A**) 4-NMBA and (**B**) 4-TNM ligands showing the different fragmentation patterns. Inset: chemical structures of 4-NMBA and 4-TNM.

The synthesized ligands (4-NMBA and 4-TNM) were characterized by mass spectrometry (MS), as seen in Figure 1. MS with an ESI ion source was applied to determine the molecular weight of 4-NMBA and 4-TNM. In the positive ESI-MS/MS mode, the presence of an amino group causes the molecular weight to be observed as 302 Da. In addition, the sequential elimination of water residues and cleavage of the 4-PBA acid was revealed through a fragmentation pattern. 

In the case of 4-TNM, dimerization of the molecule was unavoidable due to the solvent effect, and utilizing organic solvents did not provide clear tandem, despite eliminating dimerization. The 4-TNM dimer gives 572 Da under positive ESI conditions. However, 577 Da was obtained due to the presence of amino groups. ESI-MS/MS degradation pattern revealed cleavage of water residues, coalescence of degradation products, and peaks from the dimer as expected. Nevertheless, the molecular weights of the mannose derivatives were predicted from ChemDraw (Perkins Software). The MS/MS peaks revealed possible fragmentation that agrees with ChemDraw prediction. From Figure 1, the masses of 4-NMBA and 4-TNM are close to 265.99 m/z and 270.00 m/z, indicating the cleavage of the functional group of the mannose derivatives. 

### 3.3. FTIR Characterizations 

**Figure 2:**
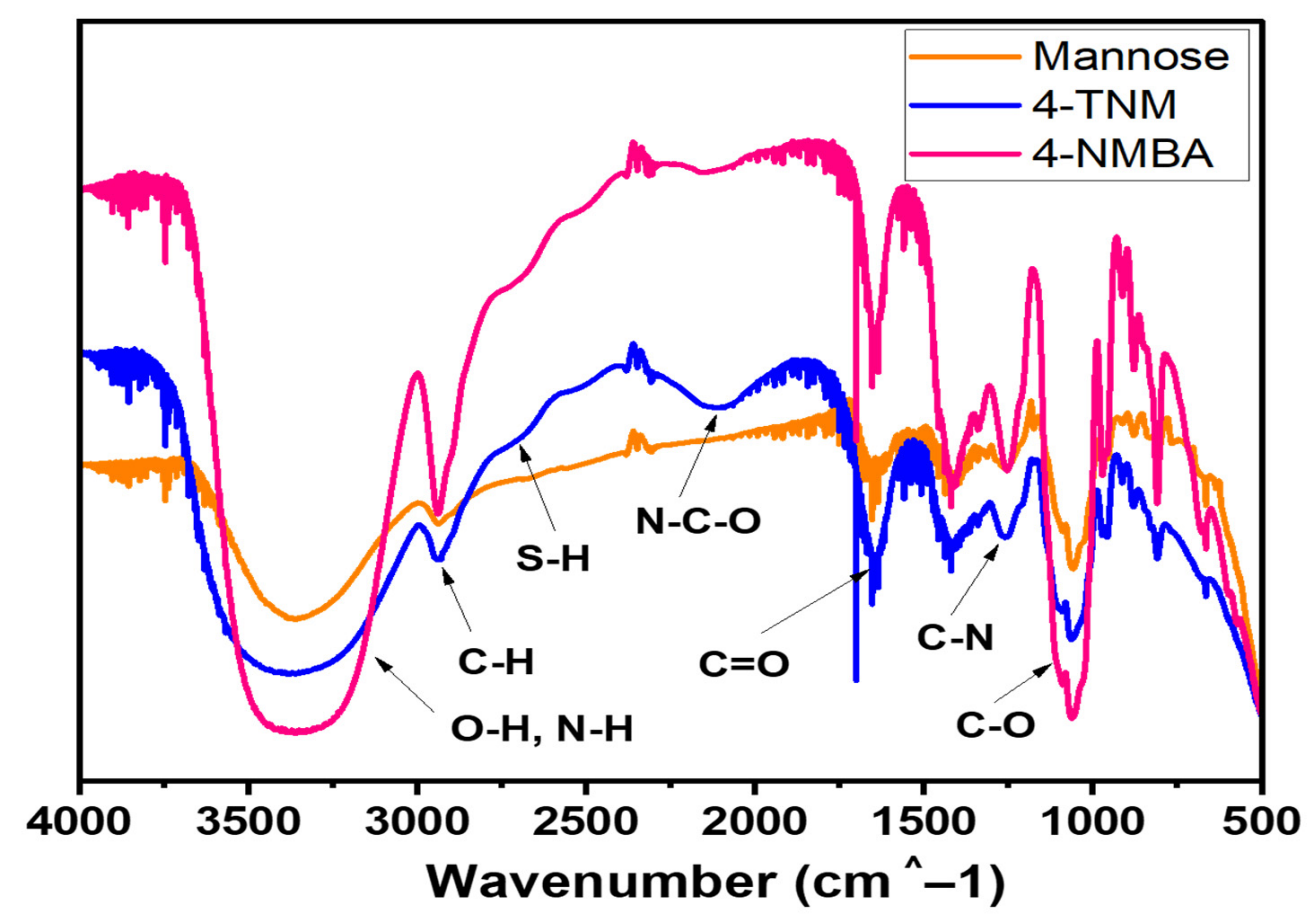
FTIR analysis of mannose and mannose derivatives (4-NMBA and 4-TNM).

FTIR analysis was conducted to determine the functional groups present in mannose and to compare them to mannose derivatives (4-NMBA and 4-TNM). In Figure 2, the characteristic vibrations at 3500 cm^−1^—(OH group), 1760 cm^−1^ (C=O), and 1716 cm^−1^ (-COOH) belong to D-(+) mannose [27]. An amide bond is expected to form in the new mannose derivatives in addition to the carboxyl groups and thiol groups as the terminal functional groups of the derivatives. The vibration bands of the carboxyl (O-H) group at 3500–3200 cm^−1^ and C–O at 1320–1210 cm^−1^ appear in both 4-NMBA and 4-TNM; however, the peak for the amide bond at 2100 cm^−1^ was present in only derivatized mannose but was absent in mannose. This observation confirmed the formation of both 4-NMBA and 4-TNM. Furthermore, the peak of the thiol group was at 2600 cm^−1^. As seen in Figure 2, the distinct peaks of carboxylic acid and thiol groups are attributed to the 4-NMBA and 4-TNM mannose derivatives, respectively. It can be concluded that the mannose derivatives were successfully synthesized. Our results agree with reports on FTIR analysis of D-(+) mannose published elsewhere [27,28].

### 3.4. SEM Characterization of Mannose Derivatives 

**Figure 3:**
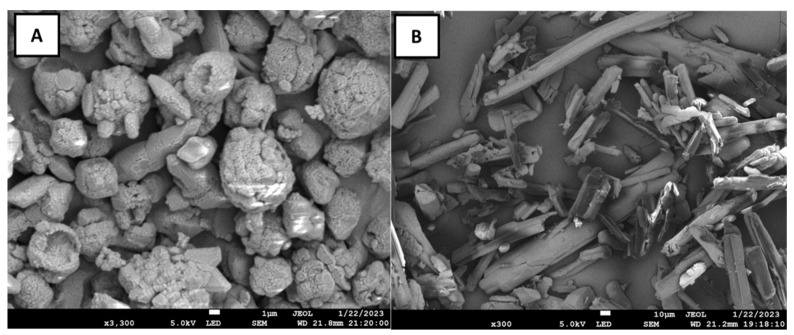
SEM characterization of mannose derivatives. (**A**) 4-NMBA and (**B**) 4-TNM.3.5. NMR Characterization.

To determine the surface morphology of mannose derivatives for FimH lectin interaction, SEM characterizations were performed using JOEL 7001F SEM. The white crystal powder of the 4-NMBA and 4-TNM were deposited on a circular aluminum stub covered with double adhesive tape. The samples were then coated in a vacuum evaporator. The visualization was performed using an accelerating voltage of 5 kV [24]. SEM images of 4-NMBA show rod and spherical shapes(Figure 3A), whereas the 4-TNM were rod-shaped as seen in Figure 3B. The formation of these observable features of the 4-NMBA and 4-TNM could be attributed to the functional groups (-COOH and -SH) of the resultant product. Shape and function play an important role in cell–cell communications. However, it is worth stating that the 4-NMBA and 4-TNM facilitate the interaction with FimH lectin of the *E. coli* owing to the knowledge that aliphatic groups and aromatic rings attached to mannose enhance its selectivity and affinity to the FimH lectin.

### 3.5. NMR Characterization

The NMR characterizations were performed to determine the amide bond formed after synthesizing the mannose derivatives. The 4-NMBA and 4-TNM showed a chemical shift of 6.7 and 7.9 attributed to the phenyl rings as compared to the shifts at 4.93 and 5.21 for only mannose. NMR data confirmed the successful synthesis of modified mannose, and the detailed characterizations are published in our work elsewhere [8].

### 3.6. Sensor Chemistry and Surface Design

The two modified mannose, 4-NMBA, and 4-TNM were synthesized by reductive amination and were applied as a sensor platform due to the interaction between mannose derivatives and the FimH lectin of type 1 pili of *E. coli*. The successful binding of *E.coli* to mannose derivatives on the surface of the quartz crystal has been demonstrated through changes in frequency and subsequent changes in mass determined by the Sauerbrey equation [22]. While other QCM-based bacteria sensors have been studied in the past, our approach is the first that uses novel mannose-derived ligands to detect *E. coli*. The sensor utilizes the 11-mecaptoundecanoic acid (MUA) linker due to its stability. Additionally, MUA is aliphatic and will interact with other aliphatic amino acids surrounding the carbohydrate-recognition domain of the FimH lectin. Furthermore, MUA was selected due to its simplified aliphatic structure with only one pKa value, and it provided good stability as a flexible linker [29]. Aliphatic groups, such as MUA, contribute to the increased affinity of mannose to FimH lectin [10]. The sensor was developed by following a stepwise modification approach. The modification of the gold crystal surface entails the following sequence. The addition of MUA is followed by the activation by the EDC/NHS, then the addition of the ligand. The addition of glycine removes the unbound ligand. Finally, the modified crystal then detects the *E. coli* (CFU/mL). It is worth noting that a baseline was established to determine and understand subsequent frequency changes upon adding the modified ligands and the bacteria during the modification and detection steps, respectively. Each modification step added mass to the crystal, which led to an altered frequency response [29]. The change in mass was determined by the Sauerbrey equation [22]. The final modification step entails the addition of mannose derivative to the gold crystal was applied to detect different concentrations of *E.coli* via FimH lectin interaction. The determined mass from the resultant frequency changes of the mannose derivatives and FimH lectin interaction have been summarized in Table 1. 

### 3.7. Detection of E. coli Using QCM Biosensor Immobilized with Mannose Derivatives

**Figure 4:**
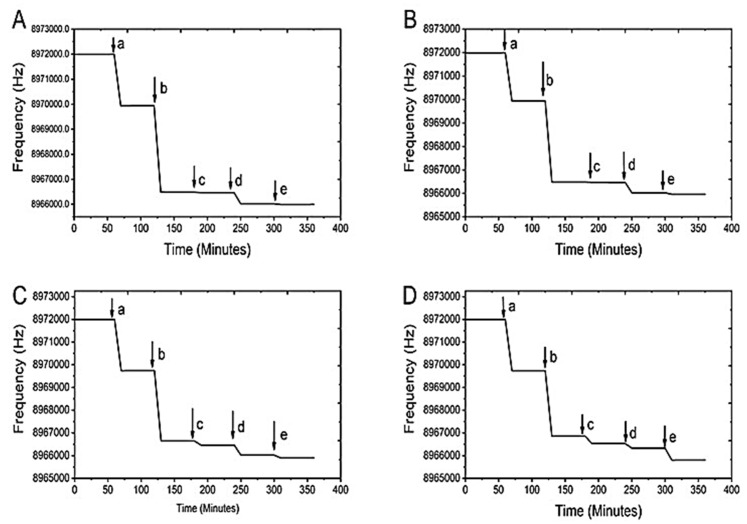
Stepwise modifications of the crystal 4-NMBA. (**A**) Represents the experiment with an *E. coli* concentration of 1 × 10^3^ CFU/mL, (**B**) 1 × 10^4^ CFU/mL, (**C**) 1 × 10^5^ CFU/mL, and (**D**) 1 × 10^6^ CFU/mL. For each graph, (a) represents the addition of the linker molecule MUA, (b) is the activation of the carboxyl group by EDC/NHS, (c) is the addition of the ligand, (d) is the addition of glycine (remove unbound ligand), and (e) is the detection of the *E. coli*.

The detection of *E. coli* via carbohydrate and lectin interaction has been demonstrated with a mannose derivative immobilized QCM sensor. The fabrication of the QCM biosensor using 4-NMBA and 4-TNM was carefully achieved through a sequential modification. The purpose of each modification was to introduce aliphatic groups to the gold crystal surface before adding derivatized mannose. The aliphatic groups increase the selectivity of mannose derivatives to the FimH lectin. Figure 4 shows the stepwise changes in the frequency of the gold crystal dependent on time for each modification step and the detection of *E.coli* over the range of 1 × 10^3^ CFU/mL to 1 × 10^6^ CFU/mL. Each graph represents a different concentration of *E. coli* tested with 4-NMBA. 

**Figure 5:**
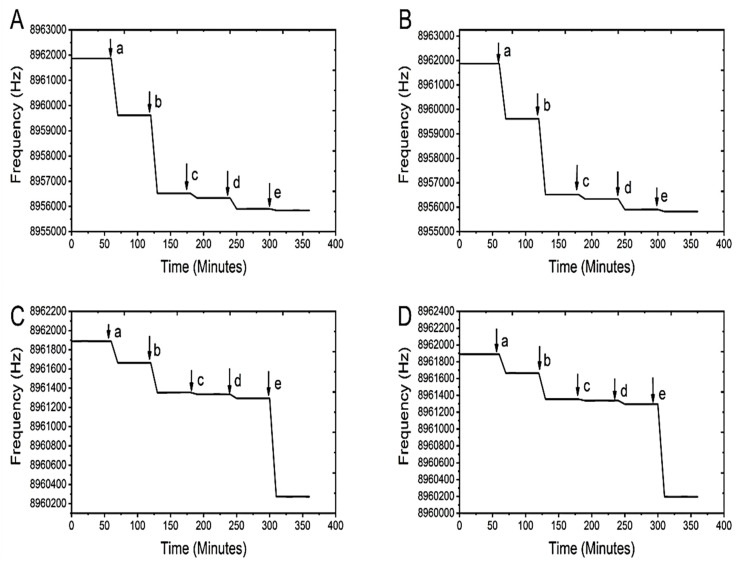
Stepwise modifications of the crystal using 4-TNM. Each plot represents the experiment with an *E. coli* concentration of (**A**) 1 × 10^3^ CFU/mL, (**B**) 1 × 10^4^ CFU/mL, (**C**) 1 × 10^5^ CFU/mL, and (**D**) 1 × 10^6^ CFU/mL. For each graph, (a) represents the addition of the linker molecule MUA, (b) represents the activation of the carboxyl group by EDC/NHS, (c) is the addition of the ligand, (d) represents the addition of glycine, and (e) is the detection of the *E. coli*.

The detectable frequency for each modification step is represented by a letter at the end of each horizontal line. Specifically, (a) shows MUA addition to the ligands, (b) shows the EDC/NHS coupling, (c) shows the addition of the mannose derivative, (d) shows the addition of glycine, and (e) shows the addition of different concentration of *E. coli*. The frequencies from each modification were converted into masses using the Sauerbrey equation [17,21]. Further calculations were performed to quantify the *E. coli* cells attached to the crystal surface. Figure 5 shows the frequency response from the immobilization of 4-TNM ligands on the QCM gold quartz crystal and its successive modification steps for the detection of *E.coli* over the concentration range of 1 × 10^3^ CFU/mL to 1 × 10^6^ CFU/mL. 

It is worth noting that there is an inverse trend between frequency and the added mass on the surface of the gold crystal. The increase in mass leads to a reduction of the crystal frequency. The difference in frequency of the quartz crystal is proportional to the mass on the surface of the crystal. After the modification steps, adding *E. coli* to the crystal resulted in a mass change of 3.796 ng for the 4-NMBA (Figure 4) and 314.2 ng for the 4-TNM (Figure 5). Assuming that one *E. coli* cell weighs 1 picogram, this equates to 3796 bacterial cells. The number of *E. coli* cells for the 4-TNM was significantly higher and can be attributed to the fact that 4-TNM has two primary amine groups [8].

**Figure 6:**
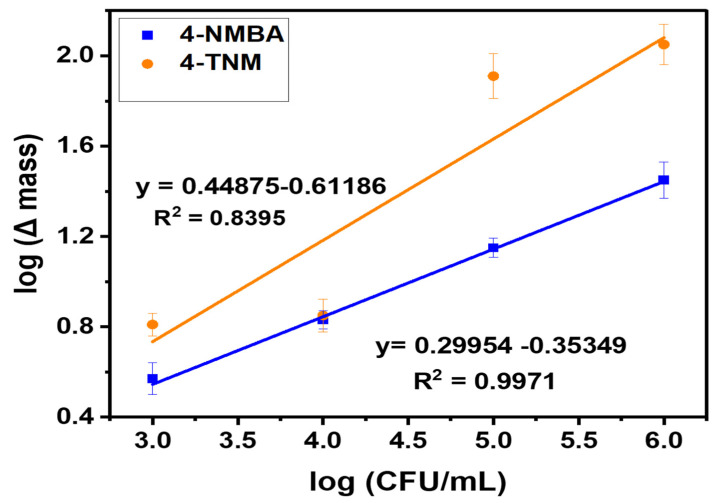
Calibration curve for 4—NMBA ligand and 4—TNM ligand. N = 3 with standard deviations of 0.023 and 0.118, respectively.

The calibration plots for 4-NMBA and 4-TNM are shown in Figure 6. The changes in mass determined from the frequencies of the detected *E. coli* were plotted against the concentrations of each ligand. From these data, the sensitivity of the 4-NMBA ligand was determined to be 2.56 × 10^−5^ ngCFU/mL, and the sensitivity of the 4-TNM ligand was determined to be 8.99 × 10^−5^ ng/mL. From the results, the interaction between mannose derivatives and the FimH lectin yielded small changes in mass [8], hence increased sensitivity as compared to the individual modification steps shown in Table 1. However, the non-linearity of the 4-TNM could be attributed to factors including the functionality of the ligand, over-oversaturation, or less uniform coating during each modification step. Experiments were performed in triplicates, with standard deviations of 0.023 and 0.118, respectively. The limit of detection was calculated to be 3.7 CFU/mL for 4-NMBA and 6.6 CFU/mL for 4-TNM. 

Table 1 summarizes the changes in mass for adding each modifying molecule to the crystal to detect different concentrations of *E. coli*. Figure S1 (in Supplementary Materials) shows the mass difference obtained for the individual modification steps in developing the QCM Biosensor. Quartz crystal microbalance has recently been used in sensing applications due to the need for accurate and ultrasensitive detection methods. For a typical quartz crystal microbalance, 1 Hertz has a lower limit of 1 nanogram of detection [30]. If the weight of one bacterium is 1 picogram, then the latter relationship explains the lowest achievable limit of bacteria detection using a QCM biosensor [30,31].

### 3.8. Performance Analysis of QCM-Based Mannose Derivative to Other Sensors

Several sensors have been reported for the detection of *E. coli.* The results from these sensors show that the sensor’s performance is based on the choice of the ligand and the mechanism of detection. A comparative analysis of this work and recent developments in bacterial sensors has been illustrated in Table 2. The mannose derivatives (4-NMBA and 4-TNM) show a superior specificity and sensitivity compared to other sensors in Table 2 owing to the low detection limit. 

### 3.9. Selectivity Sensitivity, Repeatability, and Reproducibility of Mannose Derivatives to E. coli 

**Figure 7:**
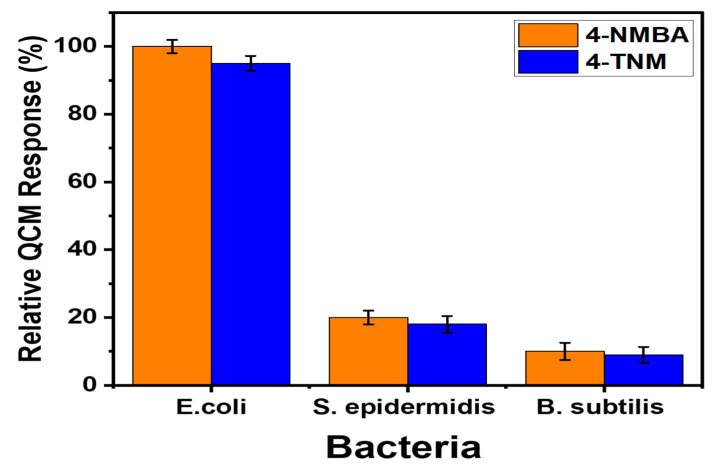
Selectivity for *E. coli* compared to *S. epidermidis* and *B. subtilis*.

Carbohydrate–lectin interactions at the surface of the host and pathogen reveal a crucial role in determining infections [30]. The selectivity and specificity of the carbohydrate–lectin interactions are necessary for the design of a reliable and robust biosensor. The detection of *E. coli* using QCM immobilized mannose derivatives is highly specific and sensitive due to mannose interaction with the FimH lectin. This selectivity study was performed on *E. coli* (model organism), *Staphylococcus epidermidis,* and *Bacillus subtilis* [38]. The choice of microorganism was based on the difference in the gram(-) and gram (+) [30]. It is expected that the bacteria with FimH lectin will bind to the mannose derivative, and the absence of FimH will lead to a low binding affinity. In Figure 7, our results showed that the mannose derivative shows 100% high specificity and sensitivity to *E. coli*, (gram(-)) due to enhanced derivatized mannose interaction with *E. coli* as compared to *S. epidermidis* (20%) and *B. subtilis* (15%). Fimbriae are short projections on the bacterial surface. FimH is an adhesive type 1 fimbria protein composed of two domains, namely an N-terminal (mannose-binding lectin domain) and a C-terminal pilin domain. FimH is expressed in enterobacteria (gram-negative *E.coli*) [30,39]. The basis of the carbohydrate–lectin interactions has led to the design of several sensors for the detection of pathogenic bacteria [40].

To promote the practical application of mannose-derivatized QCM biosensors, reproducibility, and repeatability experiments were performed following the protocol described in the experimental section above. The repeatability experiment was performed using the same gold crystal after seven successive experiments (*n* = 7). Briefly, the ligands (4-NMBA and 4-TNM) were successfully immobilized on the gold crystal along with the MUA linker molecule. The MUA was then activated by using EDC/NHS. The unbound ligand was removed by the addition of glycine. Finally, the prepared gold crystal was applied to detect *E. coli*. The results from the repeatability studies gave a standard deviation (SD) of 0.033 and 0.215 for 4-NMBA and 4-TNM, respectively. The reproducibility experiments (*n* = 7) were performed following a similar protocol and showed an SD of 0.023 (R^2^ = 0.9964) and 0.118 (R^2^ = 0.852) for 4-NMBA and 4-TNM, respectively. These results show the suitable application of the QCM biosensor for the detection of bacteria that express FimH lectins. 

## 4. Conclusions

We have successfully designed and tested a new method for detecting *E. coli* by quartz crystal microbalance immobilized with mannose derivative via the high-affinity interaction between mannose and the FimH lectin. Two mannose derivatives (4-NMBA and 4-TNM) were synthesized via reductive amination and applied in the QCM biosensor fabrication. After the QCM experimentation, the 4-NMBA yielded a mass of 3.796 ng when it adhered to *E. coli* on a modified crystal. The 4-TNM ligand was synthesized from 4-aminophenyl disulfide and mannose, resulting in a mass of 314.2 ng when it interacted with *E. coli.* The sensitivity of the 4-NMBA ligand was determined to be 2.56 × 10^−5^ ng/mL, and the sensitivity of the 4-TNM ligand was determined to be 8.99 × 10^−5^ ng/mL. This study shows the suitability of the QCM biosensors to detect pathogenic bacteria as demonstrated in several reports.

## Figures and Tables

**Table 1:** Determined masses from each modification step of mannose-derived QCM biosensor to detect *E. coli.*

Changes in Mass Due to the Addition of Ligands and Detection of *E. coli*								
Sequential Modification of Quartz Crystal	4-NMBA (ng)				4-TNM (ng)			
1. Addition of MUA	225.7	225.6	247.5	246.8	245.6	248.1	248.6	247.3
2. Activation via EDC/NHS	49.68	379.1	340.3	316.9	339.8	341.0	340.1	341.1
3. Addition of ligand	1.495	1.379	19.73	35.79	20.71	19.72	19.43	18.68
4. Glycine	4.890	49.02	47.66	21.57	43.58	57.62	47.40	47.19
5. Detection of *E. coli*	3.796	7.156	14.00	32.72	6.606	9.061	11.22	12.08

**Table 2:** Comparative analysis of mannose-derived QCM biosensor to other sensors.

Methodology	LOD (CFU/mL)	Linear Range (CFU/mL)	References
Electrochemical biosensor	48	10^0^–10^7^	Lin et al. [32]
Optical biosensor	100	-	Pen et al. [33]
QCM aptasensor	1.46 × 103	10^2^–10^5^	Yu et al. [18]
Electrochemical biosensor	78	10^2^–10^6^	Zhang et al. [34]
SPR plastic optical fiber	10^4^–10^8^	-	Wandermur et al. [35]
SPR microcontact cell imprinting	1.54 × 10^6^	0.5–4.0 McFarland	Yilmaz et al. [17]
QCM microcontact cell imprinting	3.72 × 10^5^	0.5–3.0 McFarland	Yilmaz et al. [17]
Optical biosensor	10	10^0^–10^5^	Yaghubi et al. [36]
Optical biosensor	12	30–300	Suaifan et al. [37]
Lectin-based Q.C.M.	~3.7	1.0 × 10^3^–1.0 × 10^5^	This work

## Data Availability

Not Applicable.

## Supplementary Materials

The following supporting information can be downloaded at: https://www.mdpi.com/article/10.3390/bios13030337/s1, Figure S1: Individual mass changes for each modification step and detection of the pathogen for 4-PBAligand. (A) shows the addition of MUA, (B) shows the addition of EDC/NHS, (C) shows the addition of the ligand, (D) shows the addition of glycine, and (E) shows the addition of 1 × 10^3^ CFU/mL *E. coli*; Figure S2: Individual mass changes for each modification step and detection of the pathogen for 4-PBA ligand. (A) shows the addition of MUA, (B) shows the addition of EDC/NHS, (C) shows the addition of the ligand, (D) shows the addition of glycine, and (E) shows the addition of 1 × 10^4^ CFU/mL *E. coli*; Figure S3: Individual mass changes for each modification step and detection of pathogen for 4-PBA ligand. (A) shows the addition of MUA, (B) shows the addition of EDC/NHS, (C) shows the addition of the ligand, (D) shows the addition of glycine, and (E) shows the addition of 1 × 10^5^ CFU/mL *E. coli*; Figure S4: Individual mass changes for each modification step and detection of pathogen for 4-PBA ligand. (A) shows the addition of MUA, (B) shows the addition of EDC/NHS, (C) shows the addition of the ligand, (D) shows the addition of glycine, and (E) shows the addition of 1 × 10^6^ CFU/mL *E. coli*; Figure S5: Individual mass changes for each modification step and detection of pathogen for 6-AHPligand. (A) shows the addition of MUA, (B) shows the addition of EDC/NHS, (C) shows the addition of the ligand, (D) shows the addition of glycine, and (E) shows the addition of 1 × 10^3^ CFU/mL *E. coli*; Figure S6: Individual mass changes for each modification step and detection of pathogen for 6-AHP ligand. (A) shows the addition of MUA, (B) shows the addition of EDC/NHS, (C) shows the addition of the ligand, (D) shows the addition of glycine, and (E) shows the addition of 1 × 10^4^ CFU/mL *E. coli*; Figure S7: Individual mass changes for each modification step and detection of pathogen for 6-AHP (A) shows the addition of MUA, (B) shows the addition of EDC/NHS, (C) shows the addition of the ligand, (D) shows the addition of glycine, and (E) shows the addition of 1 × 10^5^ CFU/mL *E. coli*; Figure S8: Individual mass changes for each modification step and detection of pathogen for 6-AHP ligand. (A) shows the addition of MUA, (B) shows the addition of EDC/NHS, (C) shows the addition of the ligand, (D) shows the addition of glycine, and (E) shows the addition of 1 × 10^6^ CFU/mL *E. coli*.
