# Supplementary material for: Sugar–Lectin Interactions for Direct and Selective Detection of Escherichia coli Bacteria Using QCM Biosensor
Source: Biosensors (Basel). 2023 Mar 3;13(3):337. doi: 10.3390/bios13030337 (PMC10046022; doi:10.3390/bios13030337)
Supplement: Supplementary file 1 [file biosensors-13-00337-s001.zip › biosensors-2156242-supplementary.pdf]

biosensors

MDPI

Supplementary Materials

Sugar–Lectin Interactions for Direct and Selective Detection of *Escherichia coli* Bacteria Using QCM Biosensor

Gaddi Eshun [1], Heather A. Crapo [2], Idris Yazgan [2] and Lauren Cronmiller [2] and Omowunmi A. Sadik [1,*]

1 Chemistry and Environmental Science, New Jersey Institute of Technology, 161 Warren Street, University Heights, Newark, NJ 07102, USA
2 Department of Chemistry, Center for Research in Advanced Sensing Technologies & Environmental Sustainability (CREATES), State University of New York at Binghamton, P.O. Box 6000, Binghamton, NY 13902, USA
* Correspondence: sadik@njit.edu

Abstract: A Lectins are proteins or glycoproteins of plant, animal, or bacterial origin that bind to cell surfaces through specific carbohydrate-containing receptor sites. They have been extensively used to study the nature of specific carbohydrates. However, their applications as biosensor recognition elements have not been extensively studied. In particular, the selectivity of detection had been hindered by the nature of the ligands employed. We hereby report the synthesis of two sugar-derived ligands via reductive amination. The first ligand was synthesized from 4-(N-mannosyl) benzoic acid and conjugated to mannose and abbreviated as 4-NMBA. The other ligand, named 4-thiophenyl-N-mannose conjugate, is abbreviated as 4-TNM. The structures of the sugar-conjugated ligands were confirmed using mass spectrometry. We have used the resulting ligand to develop a gravimetric biosensor using quartz crystal microbalance by exploiting the specific and high-affinity attraction of mannose for the FimH protein of the type 1 pili of E. coli. The ligands were covalently attached to gold QCM electrodes to detect bacteria. 4-PBA resulted in a mass of 3.796 ng, or 3796 bacteria cells and 6-AHP resulted in an added mass of 314.2 ng, or 314,200 cells. In comparison to other transduction methods, this biosensor. The 4-NMBA had a limit of detection of ~4 CFU/mL with a sensitivity of 2.56 × 103 ng×CFU/mL, and 4-TNM led to a limit of detection of ~6 CFU/mL with a sensitivity of 8.99 × 10-5 ng×CFU/m. By introducing hydrophobic groups to mannose via reductive amination, we have increased both the affinity and selectivity of mannose to E.coli. Overall, this biosensor is comparable to fluorescence detection. It provides a remarkable sensitivity for the enumeration of bacteria compared to impedance spectroscopy, voltammetric techniques, and other piezo-electric biosensors for the same analytes while providing an inexpensive alternative to traditional methods. Similar technology could be fine-tuned and tweaked to target viruses.

Keywords: Quartz Crystal Microbalance, Sensor, Bacterial enumeration, ligands, E. coli, FimH, selectivity

Citation: Eshun, G.; Crapo, H.A.; Yazgan, I.; Cronmiller, L. Sugar–Lectin Interactions for Direct and Selective Detection of *Escherichia coli* Bacteria Using QCM Biosensor. *Biosensors* **2023**, *13*, 337. https://doi.org/10.3390/bios13030337

Received: 27 December 2022
Revised: 14 February 2023
Accepted: 24 February 2023
Published: 3 March 2023

Copyright: © 2023 by the authors. Licensee MDPI, Basel, Switzerland. This article is an open access article distributed under the terms and conditions of the Creative Commons Attribution (CC BY) license (https://creativecommons.org/licenses/by/4.0/).

The supplementary data describes the detection of E.coli using reactants for 4-NMBA and 4-TNM via QCM biosensor. 4-NMBA was synthesized with 4-PBA (para aminobenzoic acid) and mannose , while the 4-TNM was synthesized with 6-AHP (4-aminophenyl disulfide). The detailed QCM results for using the 4-PBA and 6-AHP are shown below. It was concluded that mannose derivatives binds effectively to e.coli compared to 4-PBA and 6-AHP.

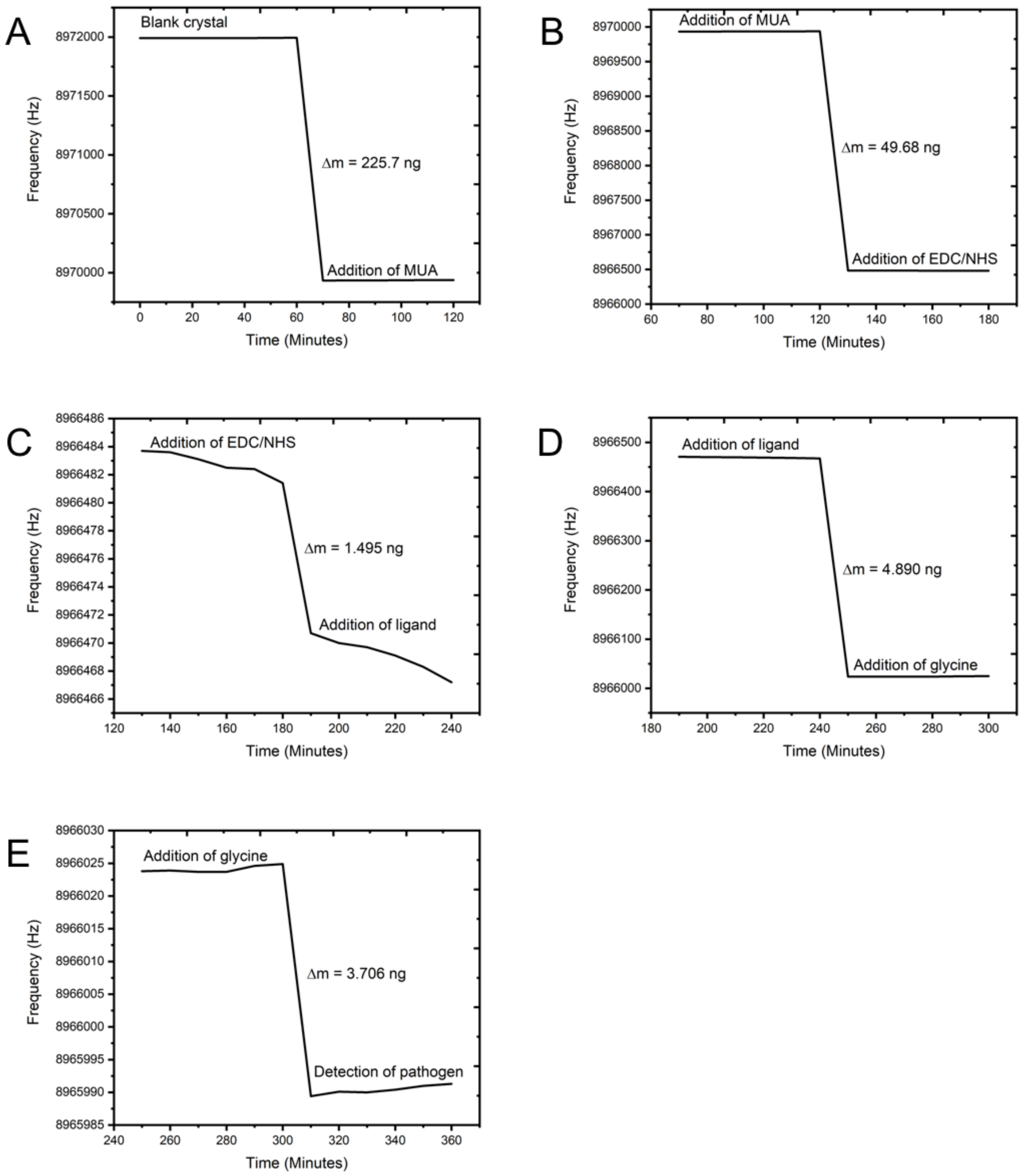

Figure S1. Individual mass changes for each modification step and detection of pathogen for 4-PBAligand. (A) shows the addition of MUA, (B) shows the addition of EDC/NHS, (C) shows the addition of the ligand, (D) shows the addition of glycine, and (E) shows the addition of 1 × 10^3 CFU/mL *E. coli*.

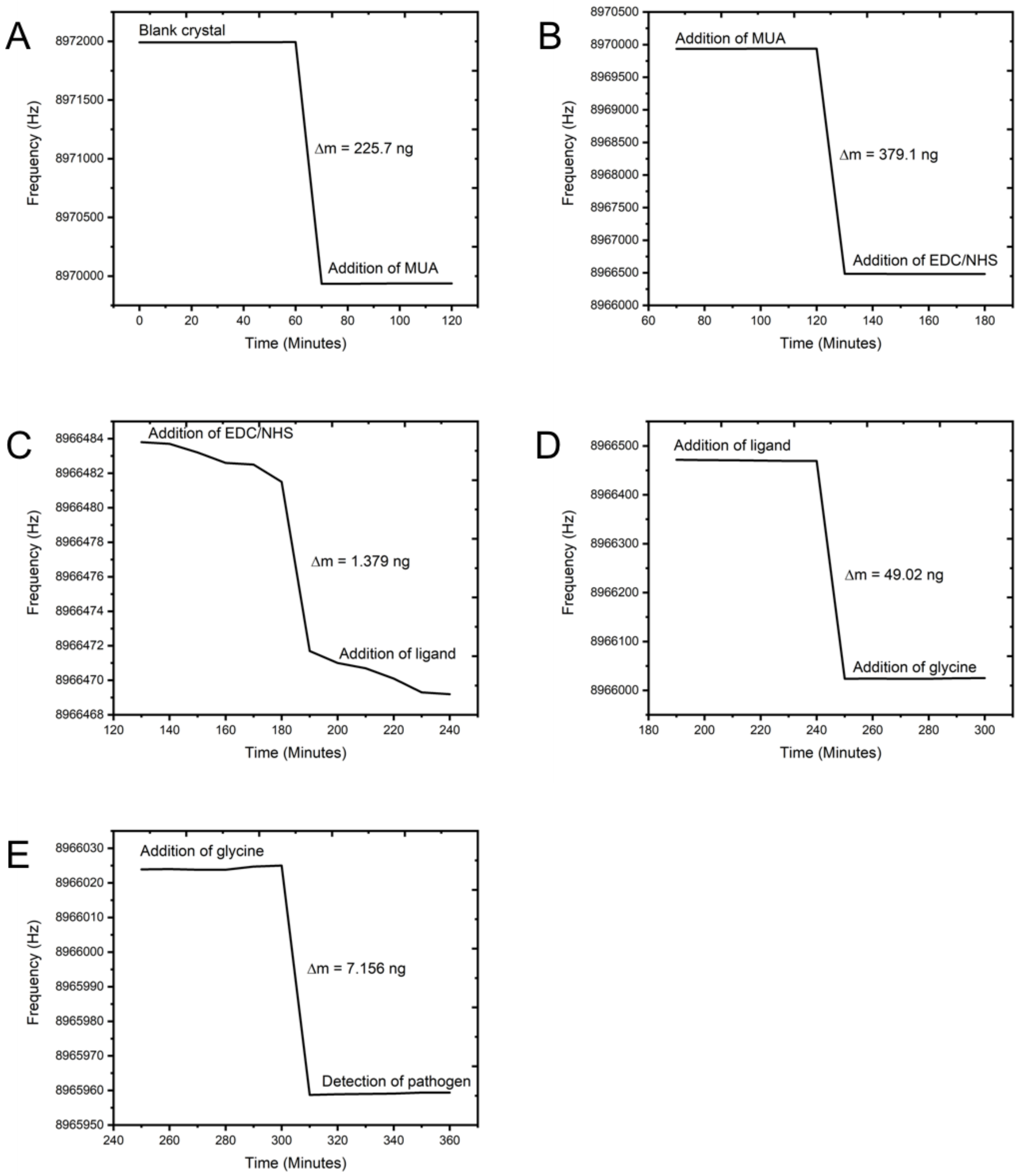

Figure S2. Individual mass changes for each modification step and detection of pathogen for 4-PBA ligand. (A) shows the addition of MUA, (B) shows the addition of EDC/NHS, (C) shows the addition of the ligand, (D) shows the addition of glycine, and (E) shows the addition of 1×10^4 CFU/mL *E. coli*.

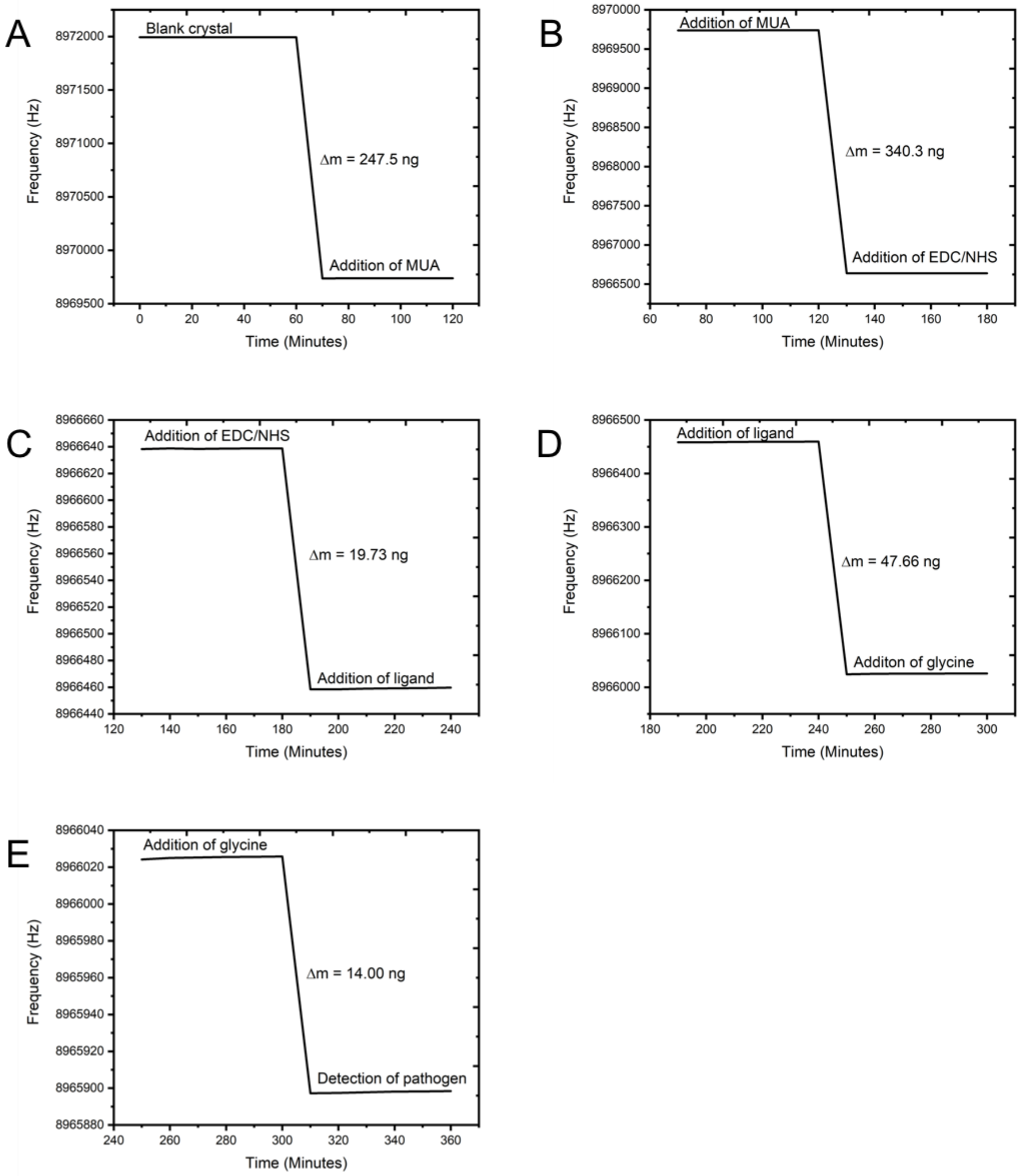

Figure S3. Individual mass changes for each modification step and detection of pathogen for 4-PBA ligand. (A) shows the addition of MUA, (B) shows the addition of EDC/NHS, (C) shows the addition of the ligand, (D) shows the addition of glycine, and (E) shows the addition of 1×10^5 CFU/mL *E. coli*.

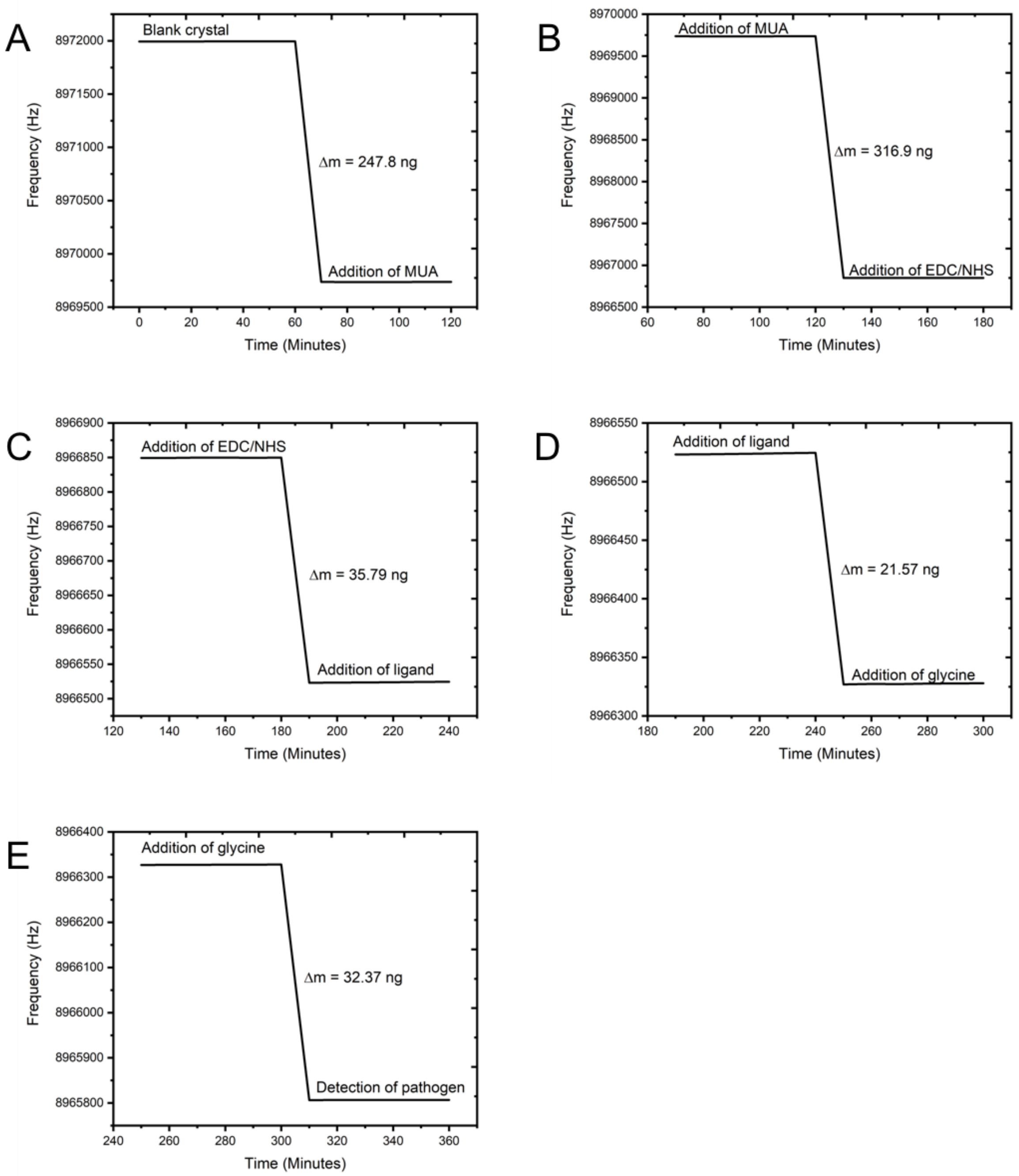

Figure S4. Individual mass changes for each modification step and detection of pathogen for 4-PBA ligand. (A) shows the addition of MUA, (B) shows the addition of EDC/NHS, (C) shows the addition of the ligand, (D) shows the addition of glycine, and (E) shows the addition of 1×10^6 CFU/mL *E. coli*.

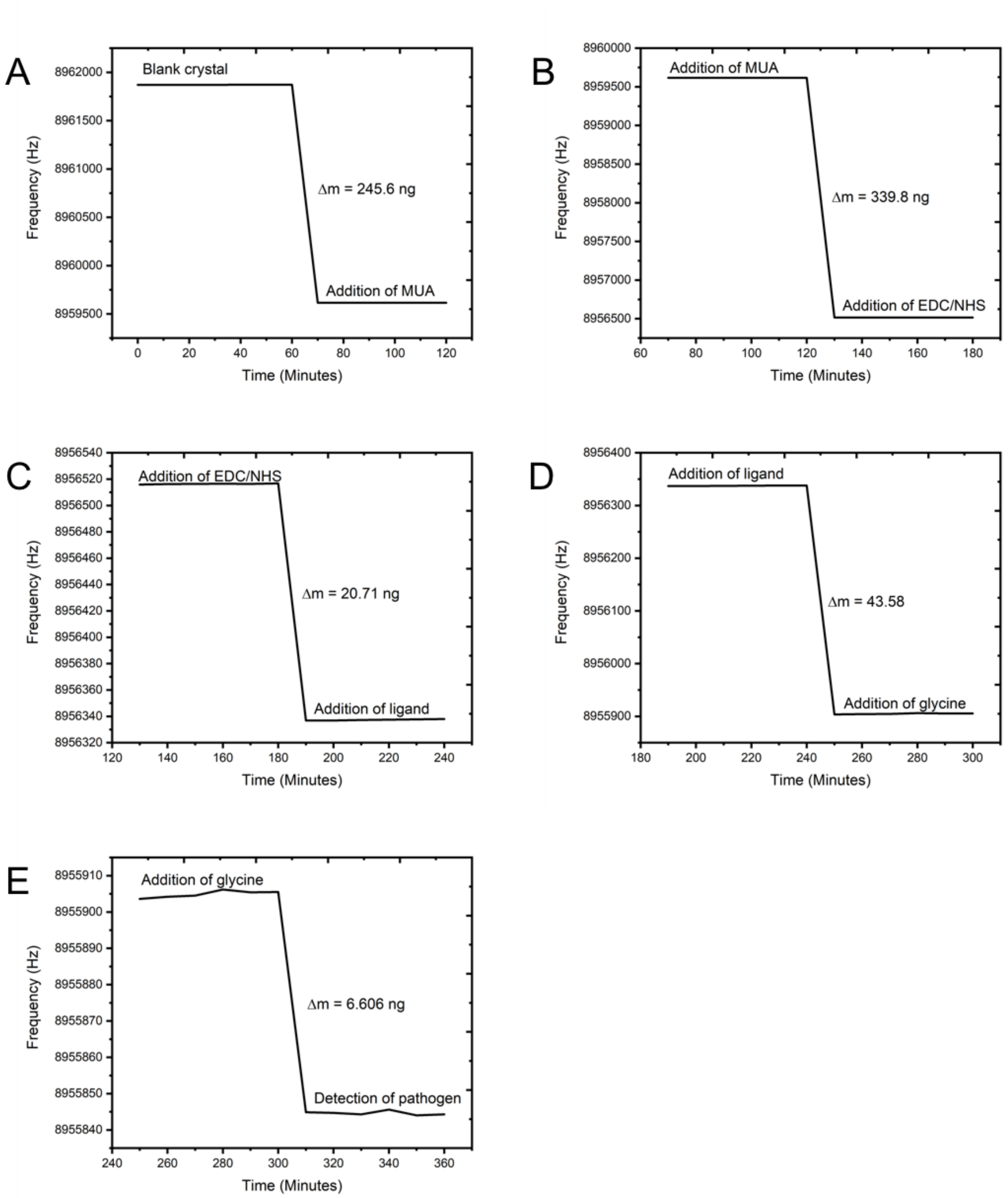

Figure S5. Individual mass changes for each modification step and detection of pathogen for 6-AHPligand. (A) shows the addition of MUA, (B) shows the addition of EDC/NHS, (C) shows the addition of the ligand, (D) shows the addition of glycine, and (E) shows the addition of 1×10^3 CFU/mL *E. coli*.

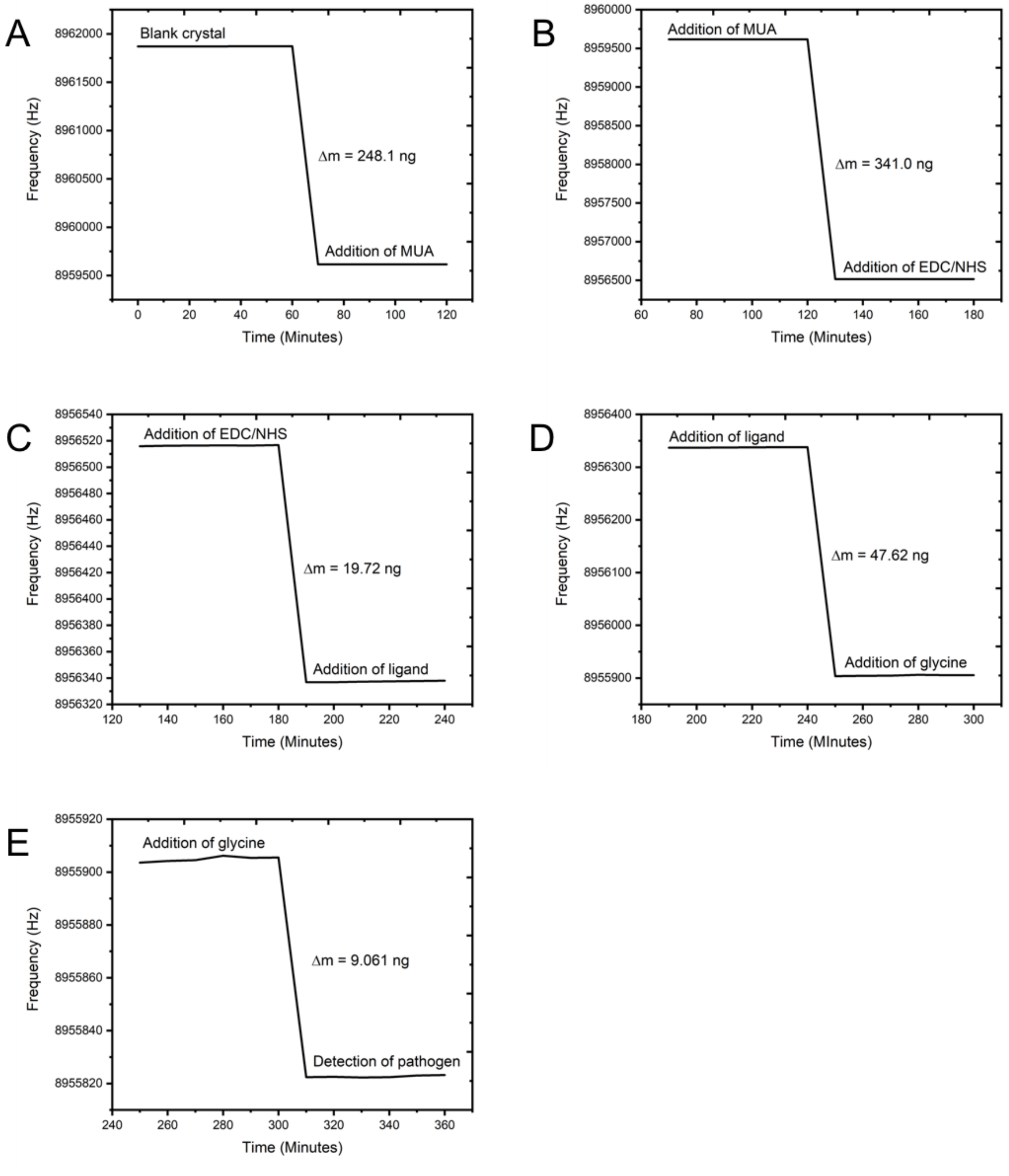

Figure S6. Individual mass changes for each modification step and detection of pathogen for 6-AHP ligand. (A) shows the addition of MUA, (B) shows the addition of EDC/NHS, (C) shows the addition of the ligand, (D) shows the addition of glycine, and (E) shows the addition of 1×10^4 CFU/mL *E. coli*.

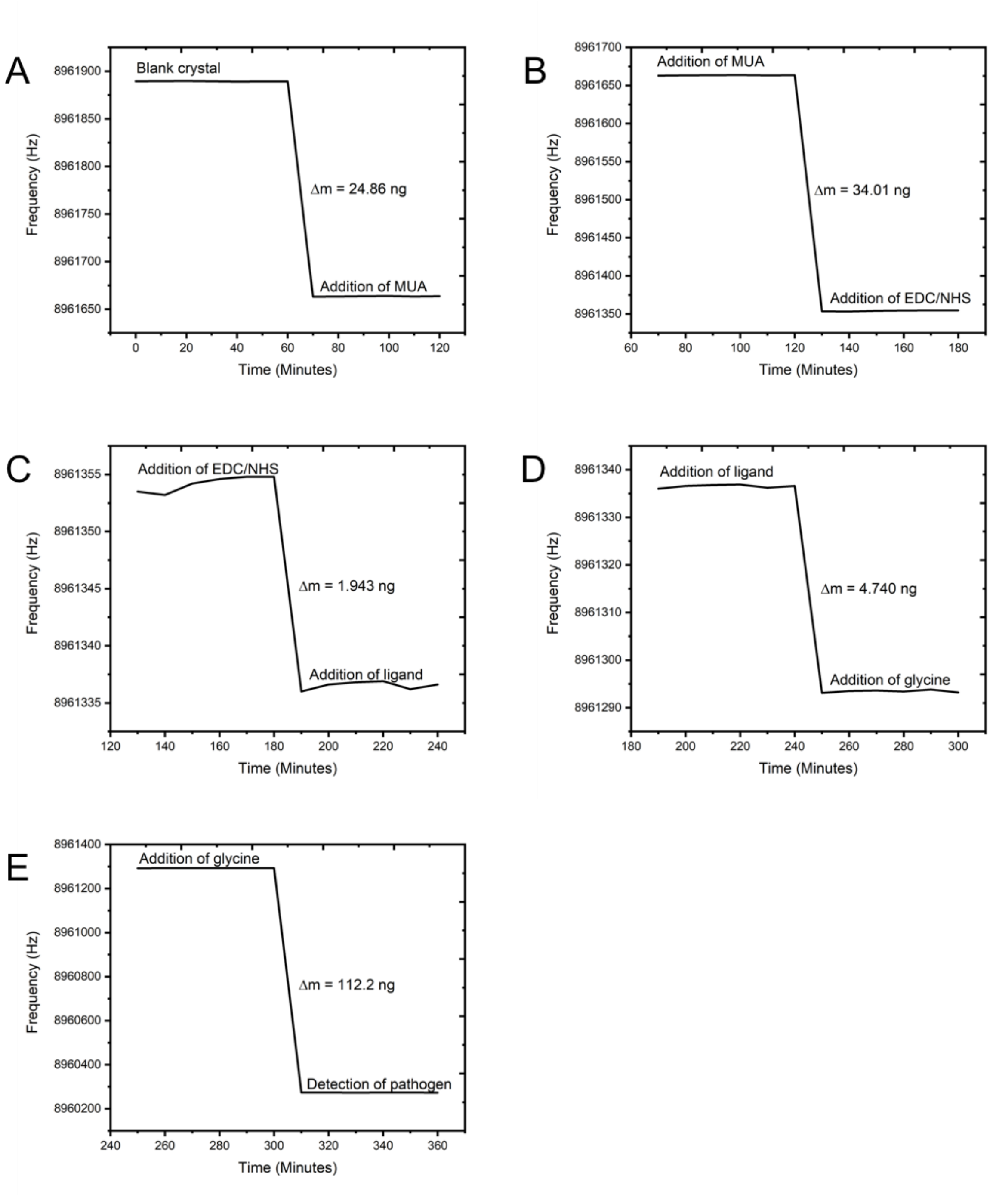

Figure S7. Individual mass changes for each modification step and detection of pathogen for 6-AHP (A) shows the addition of MUA, (B) shows the addition of EDC/NHS, (C) shows the addition of the ligand, (D) shows the addition of glycine, and (E) shows the addition of 1 × 10^5 CFU/mL *E. coli*.

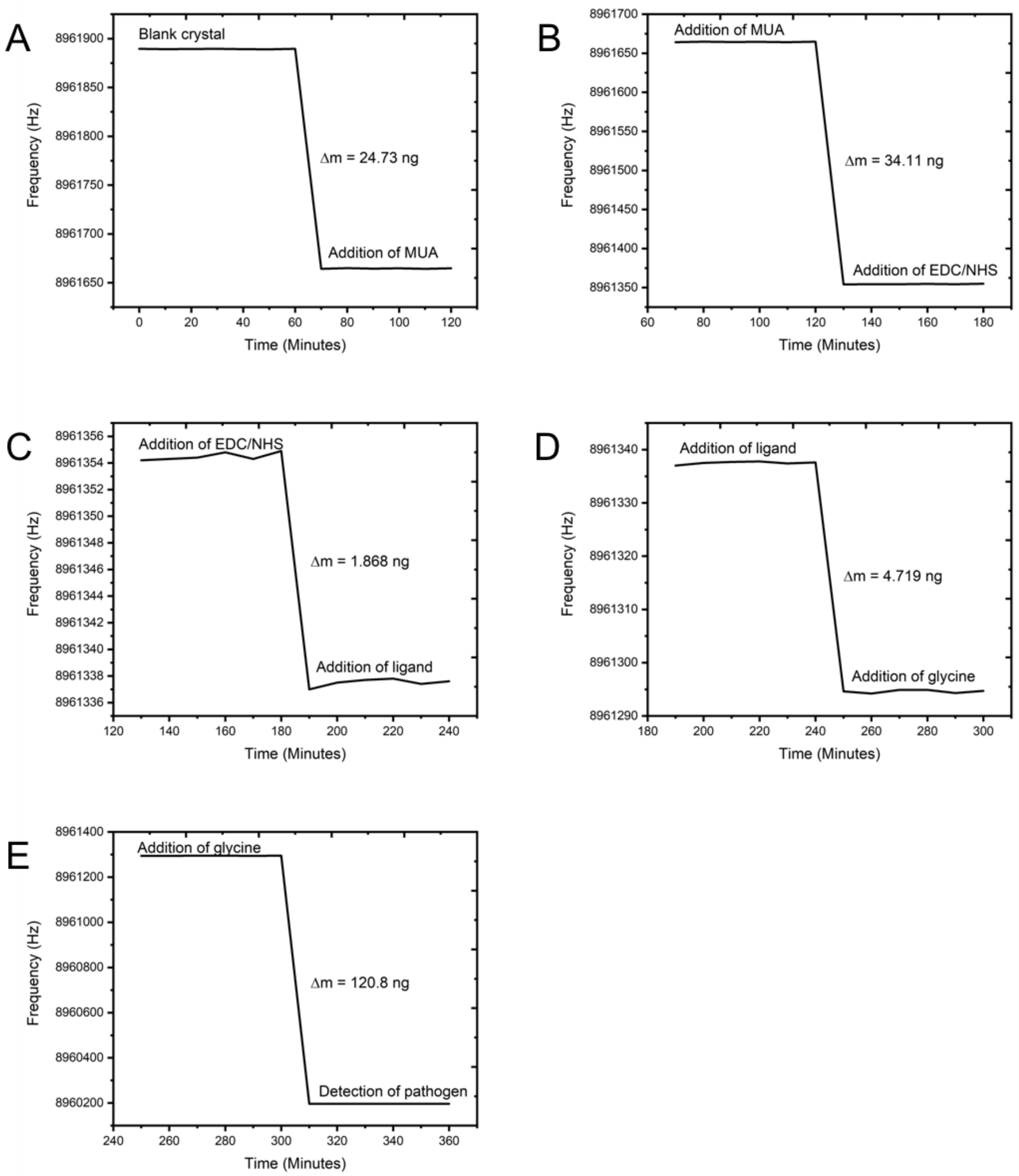

Figure S8. Individual mass changes for each modification step and detection of pathogen for 6-AHP ligand. (A) shows the addition of MUA, (B) shows the addition of EDC/NHS, (C) shows the addition of the ligand, (D) shows the addition of glycine, and (E) shows the addition of 1 × 10^6 CFU/mL *E. coli*.

Disclaimer/Publisher's Note: The statements, opinions and data contained in all publications are solely those of the individual author(s) and contributor(s) and not of MDPI and/or the editor(s). MDPI and/or the editor(s) disclaim responsibility for any injury to people or property resulting from any ideas, methods, instructions or products referred to in the content.
